# COVID-19 – An Opportunity to Redesign Health Policy Thinking

**DOI:** 10.34172/ijhpm.2020.132

**Published:** 2020-07-20

**Authors:** Joachim P. Sturmberg, Peter Tsasis, Laura Hoemeke

**Affiliations:** ^1^School of Medicine and Public Health, University of Newcastle, Callaghan, NSW, Australia.; ^2^International Society for Systems and Complexity Sciences for Health, Waitsfield, VT, USA.; ^3^School of Health Policy and Management, Faculty of Health, York University, Toronto, ON, Canada. 4; ^4^Gillings School of Global Public Health, University of North Carolina, Chapel Hill, NC, USA.

**Keywords:** Systems Thinking, Complex Adaptive Systems, Design Thinking, Health Policy Design, COVID-19, Pandemics

## Abstract

Coronavirus disease 2019 (COVID-19) dramatically unveiled the fragile state of the world’s health and social systems – the lack of emergency health crisis preparedness (under-resourced, weak leadership, strategic plans without clear lines of authority), siloed policy frameworks (focus on individual diseases and the lack of integration of health into the whole of societal activity and its impact on individual as well as community well-being and prosperity), and unclear communication (misguided rationale of policies, inconsistent interpretation of data). The net result is fear – about the disease, about risks and survival, and about economic security. We discuss the interdependencies among these domains and their emergent dynamics and emphasise the need for a robust distributed health system and for transparent communication as the basis for trust in the system. We conclude that systems thinking and complexity sciences should inform the redesign of strong health systems urgently to respond to the current health crisis and over time to build healthy, resilient, and productive communities.

## Introduction

 The ongoing coronavirus disease 2019 (COVID-19) pandemic has highlighted key global weaknesses in our health and political systems to effectively and efficiently respond to emergent challenges. Politics at large is amorphous and driven by vested-interest silos preventing policy-makers from formulating and implementing strategies to achieve goal-driven outcomes that benefit society-as-a-whole.^1-3^ Moreover at times, the lack of understanding at the highest level can lead to complacency and cause an unresponsiveness to hazards. Governments at large also lack the fundamental elements that make an organisation successful – a clear statement of purpose, clear statements of specific goals, clear statements of values, and most importantly agreed statements of organisational interactional rules, all of which are prerequisites to allow an organisation to become adaptively responsive in rapidly changing environments.^4-6^

 Understanding an organisation as a complex adaptive system entails that its functionality needs to embrace systems and complexity thinking. In other words, thinking about the fact that *everything is connected to everything else *and that perturbations in one part of the organisation effects every other is essential.

 This has important implications for policy-making (the plan needed to achieve a goal)^7,8^ as policies have been equated to experiments into a complex adaptive system. Outcomes of experiments are not predictable with certainty, and as experience has demonstrated so frequently, all policies have unintended consequences, some of which have destroyed the system-as-a-whole.^1-3,7,9^

 The COVID-19 crisis offers the opportunity to – or more precisely demands us to – collaboratively re-design^4,6,9-11^ our systems. Design starts with knowing *what we want to achieve* and then working backwards to identify what steps we need to take to get there from where we are now. We argue that adherence to such fundamentals^4-8,10,11^ is the basis for the readiness to respond to the resource demands of a pandemic, to achieve cooperation and adherence to epidemiologically proven containment strategies, and most importantly to create trust by being fully transparent – even, or especially, as new information arises.

## The Symptoms of System Failure

 Political leaders and their scientific advisors struggle to explain the nature of COVID-19, its dynamics, and the actions required to stop its spread. Mixed messages spanning from “COVID-19 is fake news,” and “It’s no more serious than the common flu,” to “Everyone is going to die,” and the daily doomsday reporting in the media leave people in a constant state of panic, distrust, and fatigue. People around the world experience new realities of daily life – home isolation and working from home (for those lucky enough), empty streets and grocery store shelves. Images of overcrowded hospitals, an exasperated health workforce, and overflowing and temporary morgues are “the new norm.” These are the signs and symptoms of our failing political, economic, and health systems.

###  Lack of Emergency Preparedness

 Despite having signed on to the World Health Organization (WHO) *International Health Regulations*^12^ to prevent the spread of diseases, many countries reduced (or even abolished) their pandemic policy and planning units, which resulted in the loss of expert knowledge, pragmatic experience, and physical resources. The neo-liberal doctrine demanding reliance on market-forces as the most efficient way of running government (and everything else) resulted in underfunding of many public goods. COVID-19 uncovered not only the lack of strategic planning, but also the massive under-resourcing of health systems.

###  Siloed Policy Frameworks 

 National governments necessarily must subdivide the organisation of their operations into various domains. These are then further subdivided into smaller divisions and even smaller groups. Each group has its own limited focus, as has every division and domain. National systems of governance lack interconnectivity; the net result is a series of self-protecting silos that insufficiently communicate and collaborate at every scale of organisation.^13^ Unsurprisingly, misperceptions and errors are not detected (in time) and become entrenched, resulting in a reinforcing loop of competition and mistrust which perpetuates the system’s instability.

###  Ad-hoc Communication

 Communication by political and scientific leaders has been slow, evasive, and repeatedly contradictory^[1]^; an additional sign of un/under-preparedness, lack of leadership, and lack of lines of authority and responsibilities in the case of a pandemic (or any other natural) crisis. Reporting of data without reference frames is misleading and distorting (eg, total number of affected/death without reference to population size/numbers tested/numbers of positive tests). Such misrepresentation invariably leads to confusion, fear, and distrust, which in turn prevent people from adhering to epidemiologically proven containment strategies. Even though uncertainty is among the inherent characteristics of pandemics, communication should be clear, and decisions made quickly based on local conditions and short time period predictions.

## A Systemic Understanding of the COVID-19 Crisis

 These symptoms of system failure are but three of the observable outcomes of our (lack of) “emergency preparedness systems.” They allow an analysis of the relationships between and dynamics among the agents that describe *societal systems-as-a-whole*. Systems as **“***whole[s] consisting of two or more parts (1) each of which can affect the performance or properties of the whole, (2) none of which can have an independent effect on the whole, and (3) no subgroup of which can have an independent effect on the whole.”*^14^

###  Infectious Disease Dynamics

 The dynamics of a pandemic are typically modelled by *Susceptible to the disease, Infective/Infected, and Recovered* (SIR) models. Each of the different states – susceptible, infected, recovered – can be configured with parameters that define the characteristics of the virus (incubation period, ease of transmission, severity of disease), environment (weather, social media impact), and characteristics of the person/population/society (susceptibility, age distribution, contact pattern, general health and resilience, socio-economic status).

 Severe acute respiratory syndrome coronavirus 2 (SARS-CoV-2) is a spontaneous mutation responsible for the emergence of COVID-19 disease.^15^ The degree of cross-immunity of SARS-CoV-2 with other betacoronaviruses responsible for flu-like illnesses is unclear, hence one must assume that the whole community might be susceptible to the infection.^16^ Without population-wide and continued testing, true incidence, prevalence, and fatality rates are unknown. Limited testing makes all data vulnerable to a severity bias, especially because many cases are aymptomatic.^17^ Our limited data indicate that between 40%-80% of people infected show no or only minor symptoms,^18-20^ but that those with multiple morbidities – at any age – are much more likely to develop severe disease requiring hospitalisation, including intensive care unit care.^21^

###  Dynamics on the Health System

 This basic SIR type model provides the expected number of patients in each state of the infection cycle at any point in time. These data can be put into a “health service model” to determine the most likely impacts on the health system as-a-whole and provide information about resource needs.

 Modelling has indicated that health systems-as-a-whole would be severely limited in their ability to cope with a sudden rise in demand, as indeed seen throughout Italy and New York City. Primary care has been impacted as much as secondary and tertiary care sectors. Modelling also highlighted the high likelihood of a large number of patients, ill with COVID-19 or other diseases, failing to get the right care, which inevitably has been associated with a higher likelihood of mortality than otherwise might have been the case (Figure).

**Figure:**
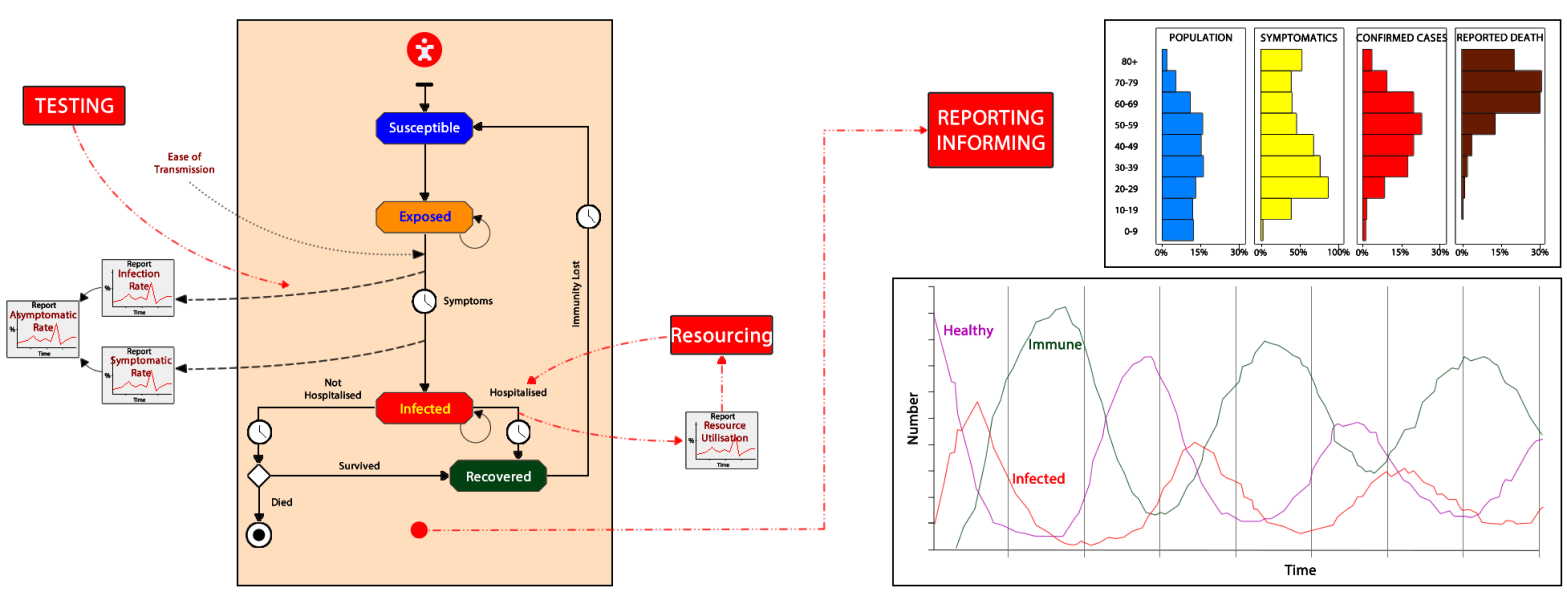
Basic Epidemiology Model, and its Potential to Contribute to the Transparency of Communication. Note: Exposed in this modified SIR model indicates the latent period during which one is infected but not infective. Abbreviation: SIR, Susceptible to the disease, Infective/Infected, and Recovered.

###  Dynamics of Crisis Management

 A crisis is a chaotic state – while many things happen simultaneously there seems to be no linkage between activities. Two approaches can be used to stabilise a crisis – implement one measure at a time and observe if linkages emerge that then can be worked with to further stabilise the situation (akin to emergency and intensive care crisis interventions), or enact authoritarian interventions that “stop everything” to allow an assessment of what might have happened (akin to a pathologist’s post-mortem examination).^22^ The former requires adaptive leadership (forward and action orientation) whereas the latter is managerialist (control focused on the *status quo*).^23^ Effective crisis leaders know how to seamlessly move back and forth between managerial and adaptive leadership as more information comes to light and as the *status quo* changes (Table 1 illustrates different leadership approaches and their un/intended consequences).

**Table 1:** Illustration of Different Leadership Approaches to the COVID-19 Pandemic

**Country**	**Leadership Approach**	**Outcomes **	**Infection and Mortality Rates (As of July 10, 2020)**
South Korea	Early widespread population testing and social isolation	Early success in suppressing spread Mid-June – spike in infections related to a single person with extensive contacts in the night life scene of Seoul Night life venues closed indefinitely	Confirmed 13 293 Death 287
Italy	Late testing of suspected sick cases, late social isolation	Largely geographically limited to the northern regions High death rate, particularly amongst the elderly High death rate amongst hospital staff Virus appears to have appeared as early as 18 December, 6 weeks before the first confirmed case on 31 January	Confirmed 242 363 Death 34 926
Iceland	Extensive testing of everyone with concerns and advice of social isolation		Confirmed 1882 Death 10
Sweden	Limited social isolation, limited testing only expanded	Only country that adopted a “herd-immunity strategy” Higher death rate than neighbouring countries Less severe economic impacts Outcome of strategy cannot yet be evaluated as the pandemic is ongoing	Confirmed 74 333 Death 5550
Australia	Country-wide log-down, compulsory quarantine of overseas arrivals, testing of suspected cases and contact tracing	Rapid success in supressing spread Rushed development of a phone-based app to help with contact tracing, but app had technical problems rendering it ineffective Reoccurring disease clusters in Melbourne, VIC, Australia due to staff failures in quarantine hotels, full log-down in Melbourne re-implemented	Confirmed 9059 Death 106
USA	Lack of any form of leadership or unified approach	Promulgation of mixed message ranging from denial, ignorance, and vested interest ideology Marked shortage of PPE and respirators, hospitals in New York City overwhelmed Highest death rates in most crowded households Black Americans and Latinos are disproportionately affected by COVID-19 Social and economic disintegration, rising racism, and xenophobia	Confirmed 3 219 999 Death 135 822

Abbreviations: COVID-19, coronavirus disease 2019; PPE, personal protective equipment.

## Redesigning Health Policy Thinking

 At the end of the Enlightenment, Humboldt observed that in the living world, *everything is connected to everything else*^[2]^. This inherent interconnectivity ought to be heeded by our health and political establishment.^24^ SARS-CoV-2 tipped all nations and communities into a health crisis, and our political leadership, from a laissez-faire mentality into a panic mode. In many societies, public health officials became key political decision-makers (and sometimes political scapegoats) in shutting down most societal activity, in turn likely protecting health and saving lives, but also stalling economic activity with the risk of a prolonged global recession. The morbidity and mortality prevented by these – seemingly draconian – measures can be estimated. However, as prevention benefits are hidden, they are often undervalued or resented – a modern-day example of Rose’s prevention paradox.^25^ Yet, the long-term negative health consequences of an economic collapse also demand careful attention.

###  Thinking Differently

 Designing arises from a way of thinking^26-28^ that incorporates the need for systemic understandings and responses to resolve problems.^6^ It embraces the need for top-down provision of information as a necessary constraint to do the required bottom-up emergent work.^29^ Design thinking is premised on the notion that we only collectively can quickly find the “right,” ie, the best adapted, solution to problems entailing diverse sources of uncertainties. This approach challenges policy-makers who are quite comfortable taking time to produce solutions based on their appreciations and need to accommodate often contradictory interests and priorities from within the ivory towers of governmental departments.^2^ This is in stark contrast to what Boulton described as “*the need for policies to be ‘live,’ dynamic *[and] *able to respond to potential unintended consequences*.”^1^

 The first step involves envisioning and agreeing on where we want to be and what we want to achieve. All ideas are welcome and rapidly tested to identify their strengths and weaknesses, which ultimately avoids the trap of missing so-called unintended consequences.^30,31^

###  Leadership – Providing Direction and Supporting Solutions

 The primary role of leaders (as opposed to managers) is to maintain a collective focus on the task at hand and to facilitate the processes of problem-solving (they are not managers who implement scripts and protocols).^5^ Leadership requires the capacity to cognitively refrain from initiatives emanating from an authoritative and core management philosophy of command and control. Leaders facilitate relationships and processes to link services, resources, and people into collective action. Leadership becomes the “*management of meaning,*” not of people or activities in which the degree of effectiveness of a leader is based on the ability to manage meaning “*in such a way that individuals orient themselves to the achievement of desirable ends*”^32^ (see Table 1 for illustration).

###  Seeing the Whole

 Policy-makers must always have the system, ie, the “whole,” in mind even for problems that seem narrow and well-defined. As Ackoff pointed out, in dynamic adaptive systems, even the most sophisticated improvement of a part invariably never achieves a result unless it also improves the system as-a-whole.^14^

## Strategies for a Systemic Pandemic Response

 Insights from systems and complexity thinking will lead to better policy-making as it starts with a *system-as-a-whole* focus. The whole-of-systems focus must be understood and embraced at all times by all decision-makers throughout the system (Table 2):

- Who does what, when, and why (a strategic plan with clearly delineated authority and responsibilities that nevertheless allows adaptation in light of changes during implementation)?
- How to manage the societal consequences of pandemic restrictions on societal activities (prevent panic and economic collapse)?
- How to communicate the plan and its implications, as well as how to update on progress (understandable presentation of data in their context)?

These guiding principles must result in transparent pandemic response strategies that – as a matter of priority – achieve:

- Guidelines to attain seamless and transparent communication between all government ministries.
- Procedures to report public health events in a timely and pre-emptive manner.
- Guidelines to enhance inter-sectorial collaboration and capacity building amongst competing government sectors.

**Table 2:** Different Types of Responses to Crisis Issues

**Issue**	**Creating Fear and Resentment**	**Creating Trust and Engagement **
Emergency health crisis preparedness (under-resourced, weak leadership, strategic plans without clear lines of authority)	Mixed messages, changing messages, Vague statements, catastrophising, resource deprivation - limited tests and reagents	Transparent strategic plan, providing resources, open and clear communication
Siloed policy frameworks (focus on individual diseases, integration of health into the whole of societal activity and its impact on individual as well as community well-being and prosperity)	Misunderstanding of the importance of other disciplines other ways of thinking about the problem Lack of attention to the interconnectedness of the entire system the impact of policies in one sector on outcomes in another sector	Integration of all policy domains need to ensure maximisation, effectiveness and efficiency of a highly functional public health system
Unclear communication (rationale of policies, consistent interpretation of data)	Cumulative numbers like number of infected number who died	Transparent data presentation providing proper context % of population infected % of infected per category (no symptoms, mild disease, severe disease) % with severe disease intubated Mortality rates/age group Change in all-cause mortality Change in all-cause mortality/age group etc

## Conclusion

 The disequilibrium caused by COVID-19 brought to the forefront the lack of a society-wide understanding of its purpose and vision, making it vulnerable to all types of disruptions including: economic instability, inequality, social exclusion, mistrust, ideological conflict, and power asymmetries – all impacting health. None of these are controllable within the boundaries of nations; solutions require a global critical discourse between political leaders and the community at large as the basis for collaborative policy design and actions. Furthermore, relationships need to develop between the global and local processes – framing the local in the context of the global.

 COVID-19 has brought upon us the opportunity to redesign health policy thinking. Our viewpoint elaborates how systems and complexity thinking strategies can help to achieve change through reframing traditional ways of thinking and doing in order to stimulate new possibilities through critical debate. In doing so, we can rise to the challenge of moving from fragmentation to adaptive self-organisation, creating well-integrated, equitable and prosperous societies resilient to sudden unexpected perturbations of any kind.

## Ethical issues

 Not applicable.

## Competing interests

 Authors declare that they have no competing interests.

## Authors’ contributions

 JPS conceived the paper and wrote the first draft. All authors contributed equally to the paper and approve the manuscript.

## Authors’ affiliations

^1^School of Medicine and Public Health, University of Newcastle, Callaghan, NSW, Australia. ^2^International Society for Systems and Complexity Sciences for Health, Waitsfield, VT, USA. ^3^School of Health Policy and Management, Faculty of Health, York University, Toronto, ON, Canada. ^4^Gillings School of Global Public Health, University of North Carolina, Chapel Hill, NC, USA.

## Endnotes

 [1] This on balance reflects their own biases and concerns. When information is missing or uncertain, our brains fill it in with their own script or driving force at the core of the individual. [2] It also happens to be the first principle of ecology, also stated by Leonardo Da Vinci (Everything connects to everything else).
